# Dimethyl fumarate ameliorates hepatic inflammation in alcohol related liver disease

**DOI:** 10.1111/liv.14483

**Published:** 2020-05-06

**Authors:** Moris Sangineto, Felix Grabherr, Timon E. Adolph, Christoph Grander, Simon Reider, Nikolai Jaschke, Lisa Mayr, Julian Schwärzler, Marcello Dallio, Alexander R. Moschen, Antonio Moschetta, Carlo Sabbà, Herbert Tilg

**Affiliations:** ^1^ Department of Internal Medicine I, Gastroenterology, Hepatology, Endocrinology & Metabolism Medical University Innsbruck Innsbruck Austria; ^2^ Department of Interdisciplinary Medicine University of Bari Bari Italy; ^3^ Christian Doppler Laboratory for Mucosal Immunology Medical University Innsbruck Innsbruck Austria; ^4^ Department of Precision Medicine University of Campania “L. Vanvitelli” Naples Italy

**Keywords:** alcohol related liver disease, alcoholic steatohepatitis, dimethyl fumarate, intestinal microbiota, Kupffer cells

## Abstract

**Background & Aims:**

Alcohol‐related liver disease (ALD) comprises different liver disorders which impose a health care issue. ALD and particularly alcoholic steatohepatitis, an acute inflammatory condition, cause a substantial morbidity and mortality as effective treatment options remain elusive. Inflammation in ALD is fuelled by macrophages (Kupffer cells [KCs]) which are activated by intestinal pathogen associated molecular patterns, eg lipopolysaccharide (LPS), disseminated beyond a defective intestinal barrier. We hypothesized that the immunomodulator dimethyl‐fumarate (DMF), which is approved for the treatment of human inflammatory conditions such as multiple sclerosis or psoriasis, ameliorates the course of experimental ALD.

**Methods:**

Dimethyl‐fumarate or vehicle was orally administered to wild‐type mice receiving a Lieber‐DeCarli diet containing 5% ethanol for 15 days. Liver injury, steatosis and inflammation were evaluated by histology, biochemical‐ and immunoassays. Moreover, we investigated a direct immunosuppressive effect of DMF on KCs and explored a potential impact on ethanol‐induced intestinal barrier disruption.

**Results:**

Dimethyl‐fumarate protected against ethanol‐induced hepatic injury, steatosis and inflammation in mice. Specifically, we observed reduced hepatic triglyceride and ALT accumulation, reduced hepatic expression of inflammatory cytokines (*Tnf‐α, Il‐1β, Cxcl1*) and reduced abundance of neutrophils and macrophages in ethanol‐fed and DMF‐treated mice when compared to vehicle. DMF protected against ethanol‐induced barrier disruption and abrogated systemic LPS concentration. In addition, DMF abolished LPS‐induced cytokine responses of KCs.

**Conclusions:**

Dimethyl‐fumarate counteracts ethanol‐induced barrier dysfunction, suppresses inflammatory responses of KCs and ameliorates hepatic inflammation and steatosis, hallmarks of experimental ALD. Our data indicates that DMF treatment might be beneficial in human ALD and respective clinical trials are eagerly awaited.

## Graphical abstract

In a preclinical model of alcohol‐related liver disease (ALD), the oral administration of dimethyl fumarate (DMF), a drug already used and approved for treatment of multiple sclerosis and psoriasis, revealed to be highly effective to prevent hepatic inflammation, steatosis and liver injury. In particular, DMF directly suppresses Kupffer cell activation and counteracts alcohol‐induced gut barrier disruption, preventing lipopolysaccharide (LPS) translocation.

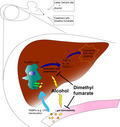

**Abbreviations**
ALD: alcohol‐related liver disease
ALT: alanine aminotransferase
Arg1: arginase1
ASH: alcoholic steatohepatitis
Cldn3: claudin3
CXCL‐1: chemokine (C‐X‐C motif) ligand‐1
DMF: dimethyl fumarate
EtOH fed: ethanol fed
FD4: fluorescein isothiocyanate‐dextran
HO‐1: Heme oxygenase‐1
IkB‐α: nuclear factor of kappa light polypeptide gene enhancer in B‐cells inhibitor, alpha
IKKα/β: Inhibitory‐κB Kinase alpha/beta
KCs: Kupffer cells
LPS: lipopolysaccharides
MCP‐1: monocyte chemoattractant protein‐1
NFκB: nuclear factor kappa‐light‐chain‐enhancer of activated B cells
ocld: occludin
p38 MAPK: P38 mitogen‐activated protein kinase
PAMPs: pathogen‐associated molecular patterns
PF: pair fed
SOCS3: suppressor of cytokine signalling3
Tjp1: tight junction protein1

**Key points**

Alcohol overconsumption is the major cause of liver disease in western countries with an important social cost, because therapeutic options are scarce. Dimethyl fumarate is an immunomodulatory drug successfully used in inflammatory disorders such as multiple sclerosis and psoriasis. In this study, dimethyl fumarate was particularly effective in reducing hepatic inflammation in a model of alcohol‐related liver disease.

## 1 INTRODUCTION

Excessive alcohol consumption and alcohol‐related liver disease (ALD) entail social, economic and individual burden worldwide. ALD represents a major cause of advanced liver disease and alcoholic steatohepatitis (ASH) with severe alcoholic hepatitis (AH) imposing a mortality rate in adults of 30%‐50% within 3 months.^1^ According to the 2018 WHO report (“Global status report on alcohol and health 2018”) 3 million deaths, ie 5.3% of all deaths worldwide, are related to alcohol consumption which occurs more frequently than diabetes‐related death.^2^ In western countries ethanol remains a main cause of liver disease,^3^ which is characterized by fatty liver development and progression to ASH and/or cirrhosis.^4^ The mechanisms of ALD progression are multifactorial, including direct ethanol toxicity on liver tissue, hepatic inflammation and alterations in the gut‐liver axis.^5^ Despite growing mechanistic understanding of ALD, treatment options remain scarce (ie corticosteroid treatment for severe AH) such that novel treatments are desperately needed.^6^

Dimethyl fumarate (DMF) is an immunomodulatory drug approved for the treatment of inflammatory diseases such as psoriasis and multiple sclerosis.^7^, ^8^ DMF is a derivative of fumarate, an intermediate substrate of the Krebs cycle which is a central process of energy production. DMF succinates kelch‐like ECH‐associated protein 1 (KEAP1), which in turn activates nuclear factor E2‐related factor 2 (Nrf‐2) to exert anti‐inflammatory and anti‐oxidant response,^9^, ^10^ while several studies also showed Nrf‐2 independent actions.^11^, ^12^, ^13^ Recently, novel mechanisms of action by DMF have been described, including the inhibition of IRAK4‐Myd88 interaction and aerobic glycolysis.^14^, ^15^ These observations are notable as activation of inflammatory cells (eg macrophages and effector lymphocytes such as Th1 and Th17 cells) requires glycolysis for differentiation and cytokine production.^16^, ^17^, ^18^ Moreover, oxidative metabolism facilitates the differentiation towards “anti‐inflammatory” subsets such as M2 macrophages and regulatory T cells (Tregs).^19^, ^20^ Consequently, DMF inhibited IL‐1β production of macrophages and reduced viability of Th1 and Th17 lymphocytes.^14^ Kupffer cells (KCs) are specialized hepatic macrophages that are critically involved in the development of ALD.^21^, ^22^ Specifically, gut derived pathogen‐associated molecular patterns (PAMPs) such as lipopolysaccharide (LPS) translocate from a leaky gut and activate KCs via toll‐like receptor 4 (TLR4),^23^, ^24^ a process that is fuelled by ethanol sensitization.^25^, ^26^ KC activation triggers production of pro‐inflammatory mediators (eg TNF‐α, IL‐1ß, IL‐6, IL‐8) that results in liver injury^23^, ^27^ and neutrophilic infiltration,^28^, ^29^ key features of ASH.^30^ Following these observations, we hypothesized that DMF ameliorates the course of ALD.

## 2 MATERIALS AND METHODS

### 2.1 Mouse experiments

C57BL/6 wild‐type (WT) mice were maintained in a specific pathogen free animal facility in Innsbruck and experiments were performed in compliance with local and national authorities (ethics approval nr BMBWF‐66.011/0155‐V/3b/2019). We used the Lieber‐DeCarli (BioServ) experimental ALD model in 8‐ to 10‐week‐old female WT mice that were exposed to increasing ethanol (EtOH) concentrations (ranging from 1% to 5%, ie 36% ethanol‐derived calories) for 15 days as described previously.^29^, ^31^, ^32^ Mice were supplemented with DMF (Sigma‐Aldrich; # 242926) at a concentration of 100 mg/kg or vehicle (methyl cellulose) by daily oral gavage for the course of the experiment, as previously reported.^14^ The Lieber‐DeCarli diet with isocaloric maltose (but without ethanol supplementation) served as control (referred to as pair‐fed). Mice were weighed every other day and drinking amounts were monitored daily. In all experiments mice were anaesthetized with xylazine (5 mg/kg) and ketamine (100 mg/kg) and blood and liver samples were collected. Plasma and liver samples were stored at −80°C or in RNAlater (Qiagen) at −20°C until further work‐up or fixed in 10%‐buffered formalin for histology.

### 2.2 Cell culture, cytokine quantification and cytotoxicity assay

A Kupffer clonal cell line (Kup5), isolated from C57/Bl6 mice, was used for in vitro experiments. Kup5 cells were cultured as previously reported.^33^ Briefly, after seeding in 12 well‐plates, Kup5 cells were polarized with LPS (1 µg/mL) (Sigma‐Aldrich) and simultaneously treated with DMF (50 µmol/L) or vehicle (DMSO) for 24 hours. Supernatant was harvested and cytokines were quantified by ELISA: IL‐1β (BD Biosciences Pharmingen), TNF‐α (BD Biosciences Pharmingen) and CXCL‐1 (R&D Systems). For western blot analysis, Kup5 stimulation with LPS ± DMF was performed for 1 hour, while mRNA was collected after 3 hours exposure. Cytotoxicity of DMF was determined by treating for 24 hours Kup5 cells, that were previously polarized with 1 µg/mL LPS for 4 hours. An LDH release assay (Roche) was used for this purpose, following manufacturer's instructions.

### 2.3 Intestinal permeability assay

Wild‐type (C57BL/6) mice (at 8‐10 weeks of age) were gavaged with DMF or vehicle for 5 days. At day six a single‐shot ethanol was administered by oral gavage, while isocaloric maltose solution served as control. After 4 hours fluorescein isothiocyanate‐dextran (FD4) (4kDa; Sigma‐Aldrich) was gavaged and 4 hours later serum FD4 fluorescence was determined by a Tecan Infinite200 plate reader.

### 2.4 Statistical analysis

GraphPad PRISM 6 was used for statistical analysis. One‐way analysis of variance followed by post hoc Bonferroni test and Spearman correlation test were used when appropriate. Results are shown as mean ± SEM. Data were considered statistically significant at *P* < .05.

Further Methods are detailed in the Supporting Information.

## 3 RESULTS

### 3.1 DMF protects against ethanol‐induced hepatic injury, inflammation and steatosis

**FIGURE 1:**
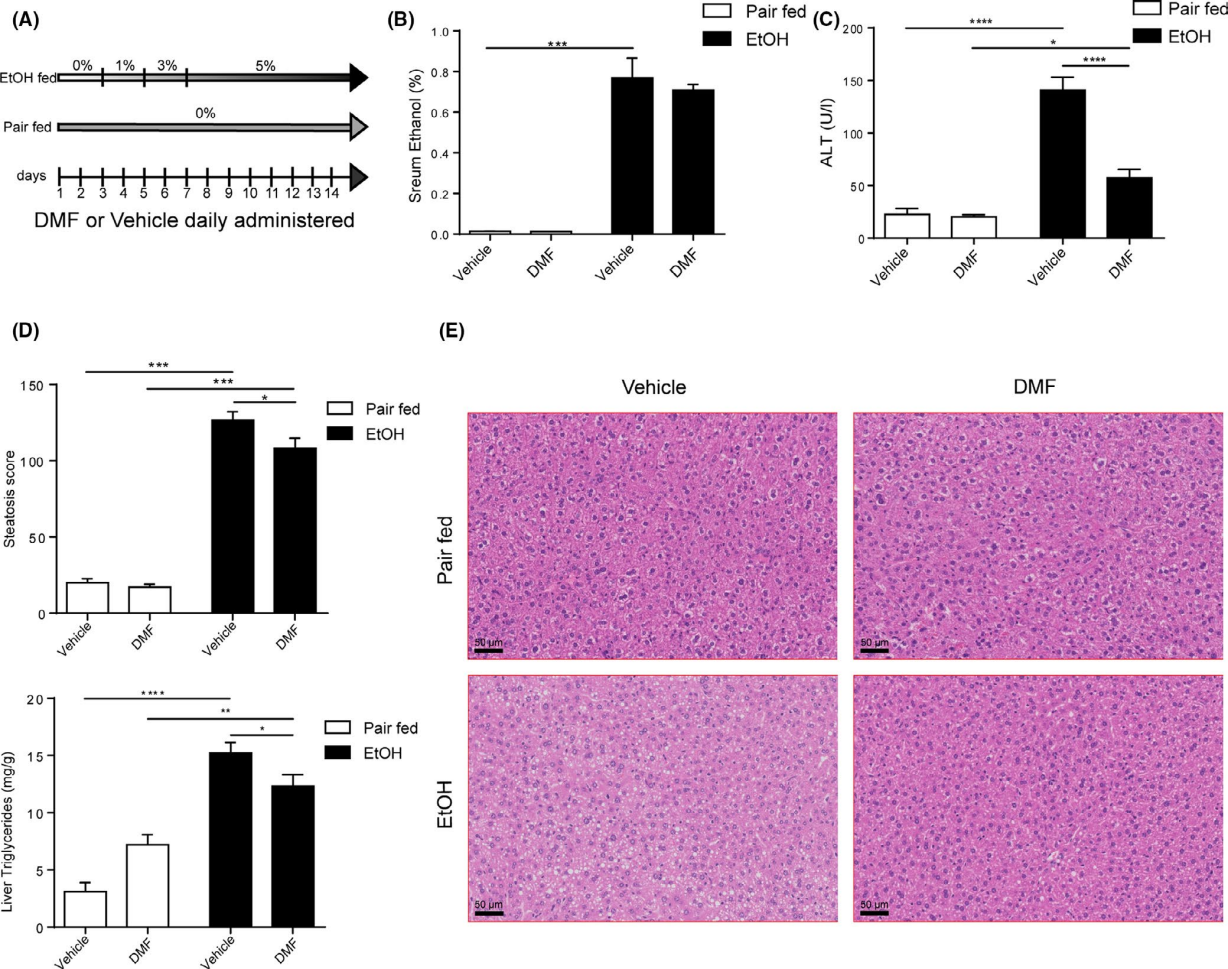
DMF ameliorated experimental ALD. A, Experimental design. B, Serum ethanol concentration (Pair fed groups = n3; EtOH groups = n6). C, Serum ALT concentration (n = 11‐14 per group). D and E, Histological determination of hepatic steatosis with representative pictures of H&E staining (Pair fed groups = n5; EtOH fed groups = n13). F, Liver triglycerides content (Pair fed groups = n5; EtOH fed groups = n12‐13). Data are expressed in mean ± SEM; *P < .05; **P < .01; ***P < .001, ****P < .0001 according to one‐way ANOVA followed by post hoc analysis (Bonferroni test). ALD, alcohol‐related liver disease; DMF, dimethyl fumarate; EtOH, ethanol

**FIGURE 2:**
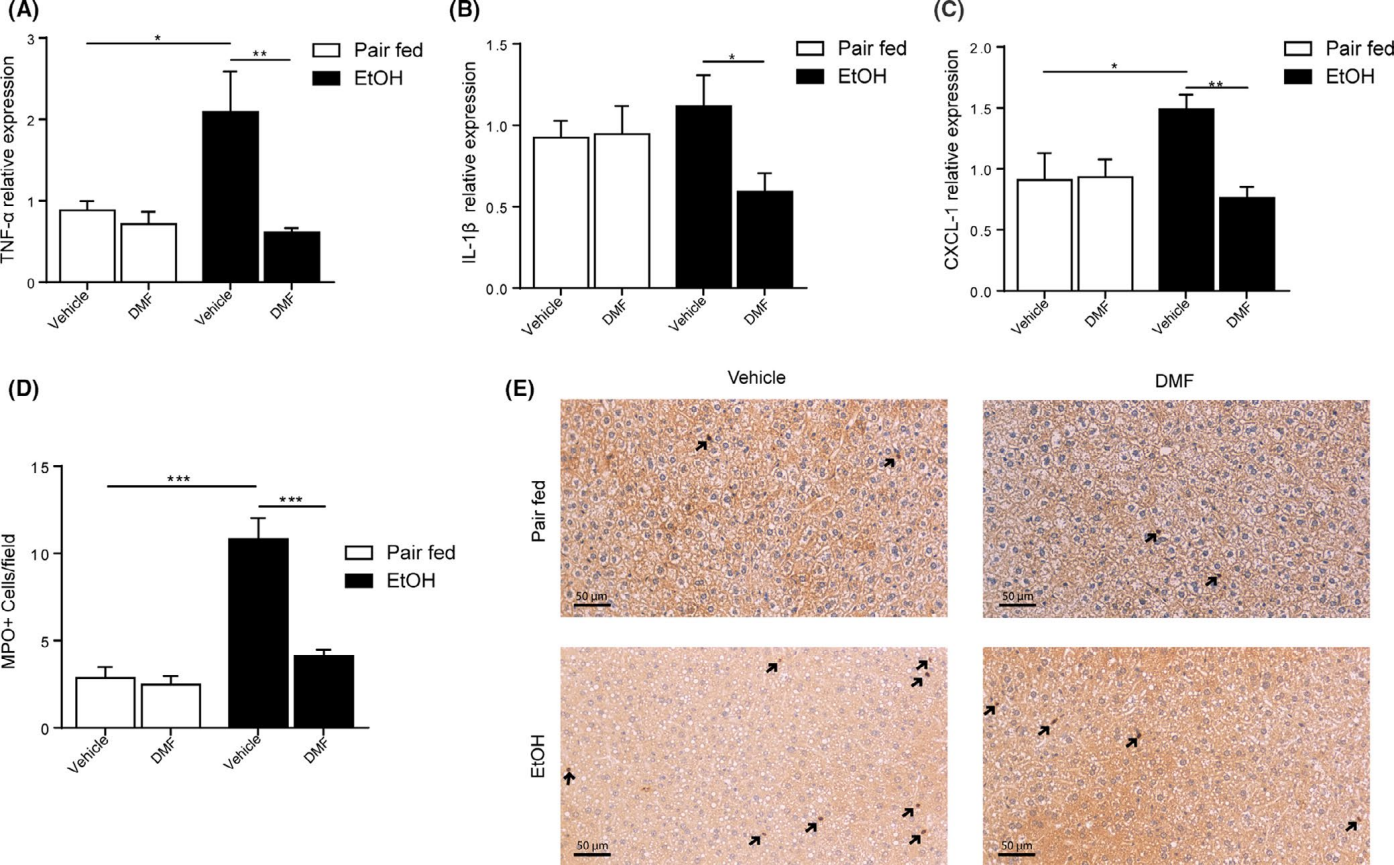
DMF dampens hepatic inflammation in mice. A, TNF‐α expression fold over *Pair fed vehicle group* determined by qPCR (n = 5‐6 per group). B, IL‐1β expression fold over *Pair fed vehicle group* determined by qPCR (n = 5‐6 per group). C, CXCL‐1 expression fold over *Pair fed vehicle group* determined by qPCR (n = 5‐6 per group). D and E, Representative pictures with quantification of neutrophils (MPO+ cells) per high power field in the liver determined by immunoreactivity (brown, indicated by black arrows; n = 5‐6 per group). Data are expressed in mean ± SEM; *P < .05; **P < .01; ***P < .001 according to one‐way ANOVA followed by post hoc analysis (Bonferroni test). DMF, dimethyl fumarate

We exposed C57BL/6 WT mice to a Lieber‐DeCarli containing 5% ethanol for 15 days and orally gavaged DMF or vehicle daily over the course of the experiment (Figure 1A). DMF‐treated mice exhibited comparable systemic ethanol concentrations compared to vehicle‐treated mice (Figure 1B). Notably, DMF protected against ethanol‐induced hepatic injury (Figure 1C) and steatosis assessed by histological and biochemical means (Figure 1D‐F; Figure S1A). Importantly, DMF treatment ameliorated ethanol‐induced hepatic inflammation indicated by reduced expression of TNF‐α, IL‐1β and the IL‐8 homologue CXCL‐1 (Figure 2A‐C **)**. In line with this, we noted reduced hepatic MPO^+^ neutrophil granulocytes count in ethanol‐exposed and DMF‐treated WT mice when compared to vehicle (Figure 2D,E). CXCL‐1 expression correlated with liver injury and MPO+ cells accumulation (Figure S1B,C **)**, suggesting that an anti‐inflammatory action of DMF accounted for the protection against ALD.

### 3.2 DMF protects against ethanol‐induced gut barrier dysfunction

**FIGURE 3:**
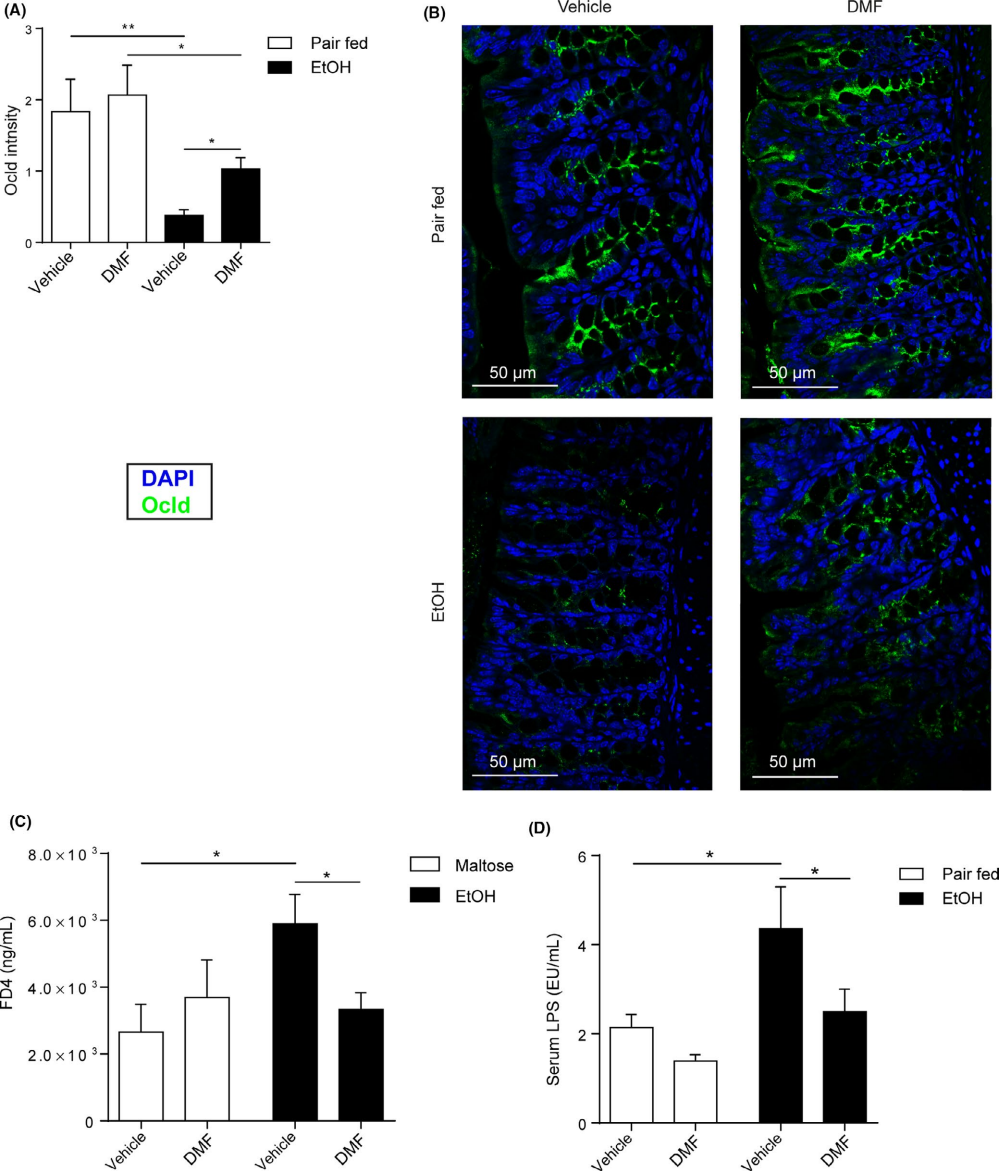
DMF improves intestinal permeability. A and B, Quantification of *occludin* immunoreactivity (green) and representative pictures of colon sections captured by confocal microscope (Pair fed groups = n3; EtOH fed groups = n5‐6). C, Serum FD4 (fluorescein isothiocyanate dextran 4) level of the in vivo gut permeability assay (Maltose groups = n3‐4; EtOH groups = n7‐8). D, Serum LPS concentration (Pair fed groups = n5‐6; EtOH groups = n6‐8). Data are expressed in mean ± SEM; *P < .05; **P < .01  according to one‐way ANOVA followed by post hoc analysis (Bonferroni test). DMF, dimethyl fumarate; EtOH, ethanol

An impaired gut barrier permits LPS translocation which activates KCs and promotes experimental ALD.^34^, ^35^, ^36^ Thus, we next studied the impact of DMF on ethanol‐induced intestinal barrier dysfunction and systemic LPS concentration. Indeed, we noted decreased *occludin* expression in ethanol‐exposed mice which was partially reversed by DMF treatment (Figure 3A,B; Figure S2A,C). In contrast, *claudin 3*, *tight‐junction protein 1*, *mucin 2* expression remained unaltered (Figure S2A,B). More importantly, DMF protected against ethanol‐induced translocation of a fluorescent dextran probe (FD4) (Figure 3C; Figure S2D) and we noted that DMF abolished ethanol‐induced LPS translocation in our ALD model (Figure 3D). These data indicated that DMF protected against a leaky gut evoked by ethanol exposure. Given the role of gut immune system, and especially monocytes, in epithelial barrier dysfunction^37^, ^38^, ^39^ we hypothesize that DMF might benefit because of its action on immune cells. Accordingly, we found that DMF significantly reduced the quantity of macrophages in colon lamina propria of ethanol fed mice (Figure S3A‐C) and diminished the expression of TNF‐α and CXCL‐1 (Figure S3D,E).

### 3.3 DMF abolishes LPS‐induced cytokine responses in KCs

**FIGURE 4:**
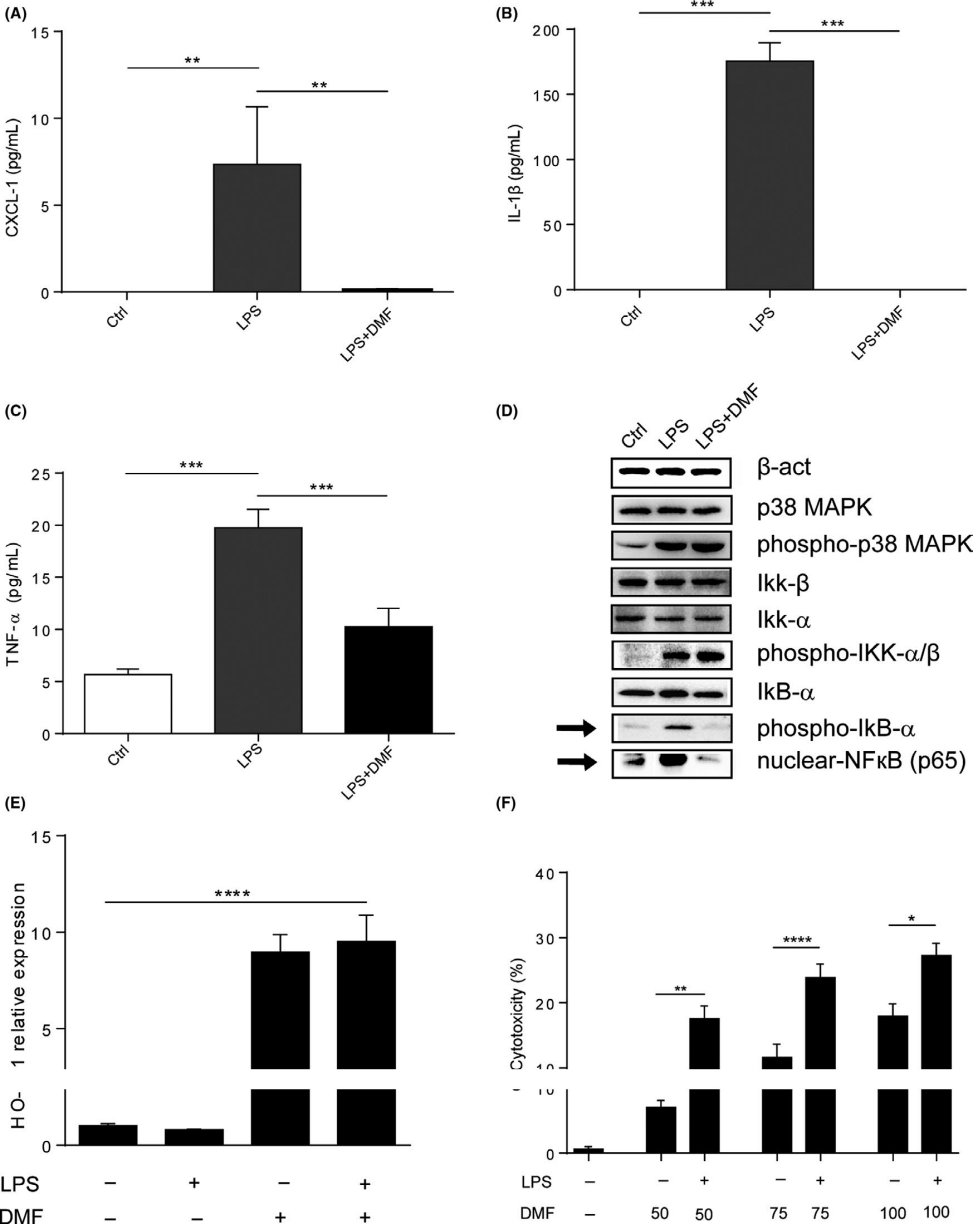
DMF inhibits M1 polarization of KCs in vitro. A‐C, Cytokines level of CXCL‐1, IL‐1β and TNF‐α in supernatants of KUP5 cells primed with LPS (1 µg/mL) and treated with DMF (50 µmol/L) for 24 h. D, Western blot analysis of typical proteins phosphorylated in TLR4‐NFκB cascade (p38, IKKα/β, IkB‐α) and nuclear level of NFκB in KUP5 cells primed with LPS (1 µg/mL) and treated with DMF (50 µmol/L) for 1 h. E, *Heme oxygenase*‐*1* (HO‐1) expression determined by qPCR in KUP5 cells primed with LPS (1 µg/mL) and treated with DMF (50 µmol/L) for 3 h. F, Cytotoxicity of different DMF concentrations (50, 75 and 100 µmol/L) on Kup5 cells previously polarized with LPS (1 µg/mL) for 4 h and then treated for 24 h; determined by LDH measurement in supernatant. For cytokines measurements and qPCR, the experiments were performed three times in triplicate. For western blot analysis and cytotoxicity assay the experiments were performed three times in duplicate. Data are expressed in mean ± SEM; *P < .05; **P < .01; ***P < .001; ****P *< .0001* according to one‐way ANOVA followed by post hoc analysis (Bonferroni test). DMF, dimethyl fumarate; LPS, lipopolysaccharide

**FIGURE 5:**
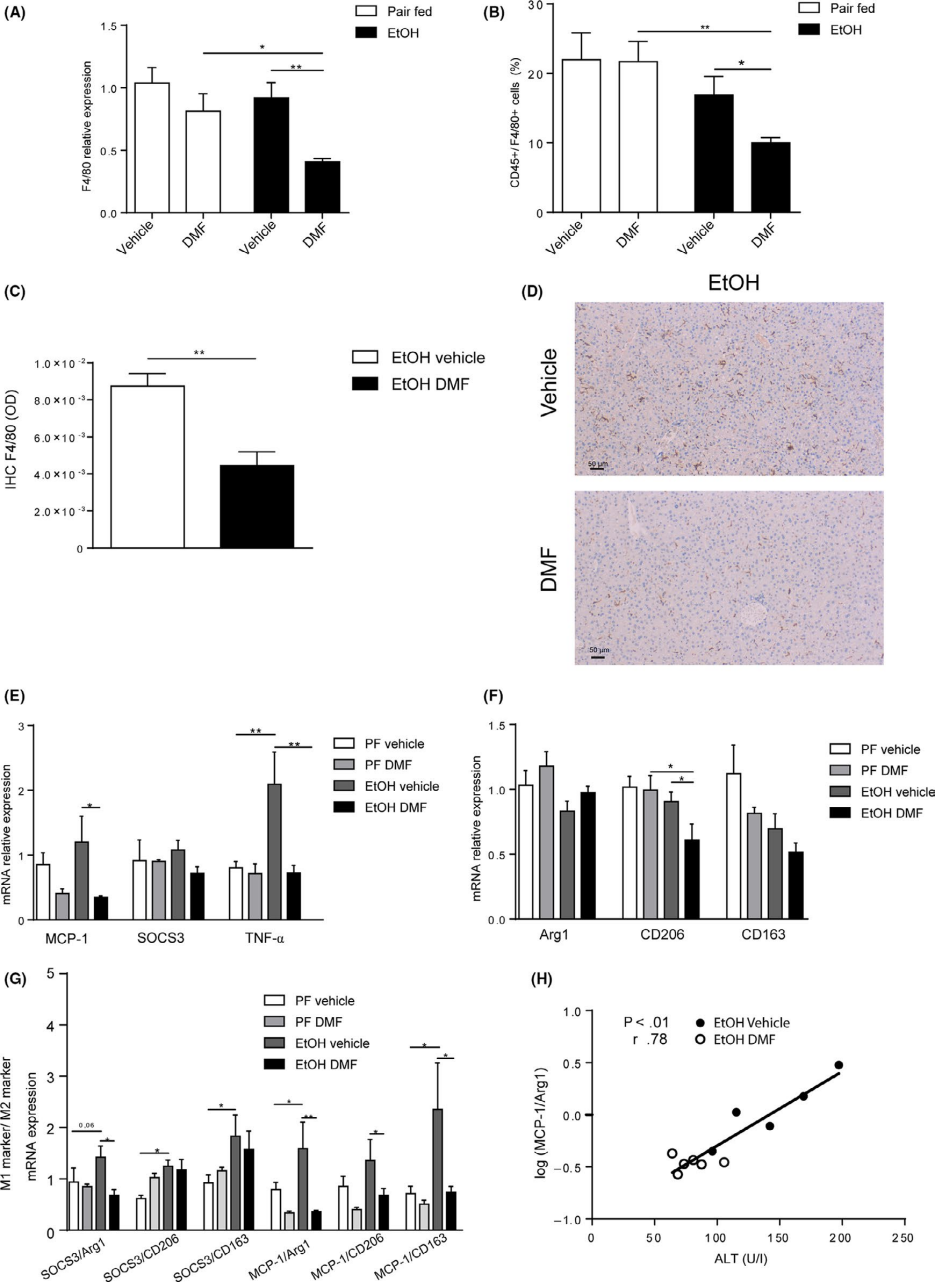
DMF impacts Kupffer cells population in experimental ALD. A, F4/80 expression fold over *Pair fed vehicle group* determined by qPCR (n = 6 per group). B, % of KCs (CD45+/ F4/80+ cells) in the liver determined by FACS analysis (Pair fed groups = n5; EtOH groups = n6‐8). C and D, Representative pictures and quantification of KCs determined by immunoreactivity to F4/80 (brown; n = 5‐6 per group). E, Hepatic expression of M1 markers (MCP‐1; SOCS‐3; TNF‐α) fold over *Pair fed vehicle group* and determined by qPCR (n = 5‐6 per group). F, Hepatic expression of M2 markers (Arg1; CD206; CD163) fold over *Pair fed vehicle group* and determined by qPCR (n = 5‐6 per group). G, Ratio of M1 markers expression (MCP‐1; SOCS3) and M2 markers expression (Arg1; CD206; CD163) (n = 5‐6 per group). H, Correlation between serum ALT and ratio of MCP‐1/Arg1 logaritmic expression in EtOH fed mice (n = 11). Data are expressed in mean ± SEM; *P < .05; **P < .01   according to one‐way ANOVA followed by post hoc analysis (Bonferroni test). Correlation is performed with Spearman test. ALD, alcohol‐related liver disease; DMF, dimethyl fumarate; EtOH, ethanol

To further explore a direct anti‐inflammatory effect of DMF in ALD, we exposed murine Kup5 KCs to LPS and monitored cytokine production with or without DMF exposure. Notably, DMF abolished LPS‐induced CXCL‐1, IL‐1β and TNFα production of KCs (Figure 4A‐C), likely by inhibiting phosphorylation of IkB‐α with consequent lower nuclear translocation of NFκB (p65) as shown by protein detection (Figure 4D). No effect was observed on phosphorylation of p38 MAPK and IKKα/β with DMF exposure (Figure 4D). In addition to this, DMF treated KCs showed a significant up‐regulation of *HO‐1* expression (Figure 4E), an important transcriptional target of Nrf2. Therefore, Nrf2 activation and NFκB inhibition could represent mechanisms by which DMF prevents LPS‐induced activation in KCs. Moreover, investigating cell injury by LDH release assay, DMF exhibited cytotoxic effects on KCs especially after LPS stimulation (Figure 4F). In line with this, DMF treatment strongly reduced hepatic KCs in ethanol‐exposed mice as demonstrated by reduced hepatic *F4/80* expression (Figure 5A). Flow cytometry quantification of CD45+/F4/80+ cells (Figure 5B) and immunolabelling of F4/80 (Figure 5C,D) confirmed this observation. Moreover, hepatic transcriptional analysis suggested a preponderant M2 (anti‐inflammatory) macrophage polarization in ethanol‐exposed WT mice that were treated with DMF (Figure 5E‐H).

## 4 DISCUSSION

Alcohol‐related liver disease and particularly its highly inflammatory condition, AH, are characterized by neutrophilic liver inflammation.^30^, ^40^, ^41^ It is conceived that gut bacteria and their PAMPs promote ALD by activation of KCs that instigate neutrophilic inflammation,^34^ which perpetuates hepatic ethanol toxicity.^25^, ^26^, ^42^ However, treatment options for ALD remain scarce which is reflected by a very high morbidity in patients with severe AH.^43^ In this study, we demonstrate that DMF, a compound that is approved for treatment of human inflammatory disorders,^7^, ^8^ exerts potent anti‐inflammatory effects and protects against hallmarks of experimental ALD. Although we noted comparable serum ethanol concentrations in DMF‐ and vehicle‐treated mice, DMF protected against ethanol‐induced hepatic injury, neutrophilic inflammation, and, to a lesser extent, steatosis. Moreover, DMF treatment protected against ethanol‐induced gut barrier dysfunction and LPS translocation, both of which represent critical drivers of ALD.^5^, ^6^, ^34^, ^36^ DMF also abolished LPS‐induced KCs cytokine production and suppressed the abundance (and probably also polarization) of hepatic KCs. These findings are notable as KCs are critical to recruit neutrophil granulocytes which perpetuate ALD by promoting ROS, hepatic injury and inflammation.^22^, ^29^, ^34^, ^41^ These findings are in line with reports, demonstrating that DMF protected against liver ischaemia/reperfusion injury with reduced expression of TNF‐α and IL‐6^44^ and a recent study indicated that DMF suppresses IL‐1β production in M1 polarized peritoneal macrophages due to inhibition of aerobic glycolysis.^14^ Moreover, DMF decreased viability of lymphocytes, especially of pro‐inflammatory Th1 and Th17 cells^14^, ^45^ and it may be plausible that DMF exerts direct inhibitory effect on neutrophil function as recently suggested in vitro.^46^ Along these lines, DMF ameliorated inflammation in a mouse colitis model.^47^

Collectively, these studies establish a potent anti‐inflammatory role of DMF and our study demonstrates a beneficial role in experimental ALD. We acknowledge that we did not investigate the effects of DMF in a liver already damaged because, considering the shortness of ALD animal models, probably a very high dosage would be needed to obtain respective therapeutic effects. Accordingly, it would be interesting to explore DMF effects in a chronic plus binge model in order to further confirm potential therapeutic efficacy. Moreover, DMF could also modulate intestinal microbial composition or function,^48^ which we did not investigate in our work. As therapeutic alternatives to treat human ALD are lacking, and as DMF exerts a favourable tolerability with barely any adverse events (and renal metabolization),^49^, ^50^ clinical studies are warranted to explore the efficacy in human ALD, and particularly severe AH.

## CONFLICT OF INTEREST

None.

## AUTHOR CONTRIBUTIONS

MS was involved in study design, conducted the experiments and wrote the paper; FG, CG, SR, NJ, LM and MD helped with experimentation. ARM, HZ, AM, TEA and CS contributed to the manuscript by conceptual and experimental means. HT designed and supervised the study and drafted the manuscript.

## Supporting information

Supplementary Material
