# Supplementary material for: Dimethyl fumarate ameliorates hepatic inflammation in alcohol related liver disease
Source: Liver Int. 2020 May 6;40(7):1610–9. doi: 10.1111/liv.14483 (PMC7383968; doi:10.1111/liv.14483)
Supplement: Supplementary file 1 — Supplementary Material [file LIV-40-1610-s001.docx]

**Dimethyl fumarate ameliorates hepatic inflammation in alcohol related liver disease**

Moris Sangineto^1,2^, Felix Grabherr^1^, Timon E. Adolph^1^, Christoph Grander^1^, Simon Reider^3,1^, Nikolai Jaschke^1^, Lisa Mayr^1^, Julian Schwärzler^1^, Marcello Dallio^4,1^, Alexander R. Moschen^3,1^, Antonio Moschetta^2^, Carlo Sabbà^2^ & Herbert Tilg^1^

^1^Department of Internal Medicine I, Gastroenterology, Hepatology, Endocrinology & Metabolism, Medical University Innsbruck, Innsbruck, Austria

^2^ Department of Interdisciplinary Medicine, University of Bari, Bari, Italy

^3^ Christian Doppler Laboratory for Mucosal Immunology, Medical University Innsbruck, Innsbruck, Austria

^4^ Department of Precision Medicine, University of Campania “L. Vanvitelli”, Naples, Italy

**Table of Content**

Supplementary Material and Methods 2

Supplementary Figures and Legends 6

References 10

**MATERIALS AND METHODS**

**ALT analysis**

Alanine aminotransferase measurement was performed in mice serum by a commercial enzymatic essay (BQ-Kit, San Diego, CA) according to manufacturer’s protocol. Briefly, 10 µL of mouse serum was added to 100 µL substrate and then the colorimetric change was measured every minute five times at 37° C. The mean of absorbances was used to quantify ALT with Tecan Infinite200 plate reader.

**Expression studies**

Tissue samples were homogenized in TRIzol® reagent (Thermo Fisher Scientific, Waltham, MA) to extract RNA. Reverse Transcription System (Thermo Fisher Scientific, Waltham, MA) was used for reverse transcription and subsequently the qPCR was performed using SybrGreen (Eurogentec, Seraing, Belgium) and Mx3000 Cycler (Stratagene California, CA). The gene expression was normalized to mouse β-actin.

**Triglycerides analysis**

Liver tissue was stored at -80° C and then homogenized in PBS, adjusting the volume to the weight, followed by 30 minutes incubation at 95° C. After a centrifugation at 12000g for 10 minutes at room temperature, the supernatant was harvested for triglycerides measurement using the appropriate reagent (Roche, Switzerland). All the procedure was conducted using vials coated with fatty-free BSA (Sigma, St. Louis, MO).

**Histology**

Liver and gut tissues were stained with haematoxylin and eosin (H&E) by the Institute of Pathology at the Medical University of Innsbruck. The h&e liver sections were used to evaluate hepatic steatosis in double blinded fashion, calculating the percentage of cells with lipid drops accumulation at different magnifications. Liver sections were also used to perform Myeloperoxidase and F4/80 immunohistochemistry. While, immune staining of F4/80 and immunofluorescence staining of occludin were performed on colon sections.

**Myeloperoxidase and F4/80 immunohistochemistry**

Liver and intestinal sections were stained as previously described[^1^](#_ENREF_1). Briefly, after deparaffinization in xylene and dehydratation in an ethanol gradient, the antigen unmasking with 2 % citrate-buffer (pH **=** 6; Vector Laboratories, Burlingame, CA) was performed. The next steps included: endogenous peroxidase inactivation with peroxidase (Dako, Santa Clara, CA) for 10 min and Protein blocking with a ready-to-use kit (MP-740; Dako, Santa Clara, CA). Primary antibodies were rabbit MPO antibodies (Dako, Santa Clara, CA) and rabbit F4/80 antibodies (Cell Signaling Technology, Inc.), while the secondary antibodies were anti-rabbit antibodies (Vector Laboratories, Burlingame, CA and Dako, Santa Clara, CA). Finally, the sections were stained with DAB (Dako, Santa Clara, CA) and counterstained with hematoxylin (Dako, Santa Clara, CA). Two blind observers counted MPO positive cells in five randomly chosen high-power fields. The F4/80 positivity was quantified with Fiji software (ImageJ, NIH) in five randomly chosen high-power fields, and subsequent conversion in optical density (OD).

**Immunofluorescence staining**

After deparaffinization of slides in xylene and dehydratation in an ethanol gradient, the antigen unmasking with 2 % citrate-buffer (pH **=** 6; Vector Laboratories, Burlingame, CA) was performed. Then a protein blocking was executed with ready-to-use kit (MP-740; Dako, Santa Clara, CA).

Primary antibodies for occludin (polyclonal rabbit antibody, Thermo Fisher Scientific, Waltham, MA) were diluted in Antibody diluent (Dako REAL, Dako, Santa Clara, CA) and incubated overnight in a humid chamber at 4°C, followed by incubation with secondary goat anti-rabbit antibodies (AF488, life technologies, Carlsbad, CA) for 1 hour. Prolong® Diamond Antifade Mountant supplemented with DAPI (4′,6′-diamidino-2-phenylindole, life technologies, Carlsbad, CA) was used to mount slides. Pictures were captured and analysed with a 340 confocal microscope (Zeiss, Oberkochen, Germany). Intensity was quantified by two blinded observers in three randomly picked fields of view.

**FACS analysis**

The hepatic left lobes were cut and incubated for 45 min at 37° C in a digestion buffer composed of Liberase in RPMI medium. Afterwards, the cell suspension was strained through a 50 μm nylon mesh, then diluted in 1% bovine serum albumin PBS and centrifuged at 30 G and 4°C for 5 min. The supernatant was centrifuged at 300G and 4°C for 5 min, and the pallet was treated with ACK buffer (150 mM NH4Cl, 10 mM KHCO3, 0.1 mM EDTA, all from Merck). After a centrifugation at 300G, 4°C for 5 min, cells were stained with Ghost Dye Violet 510 (Tonbo biosciences) for Live/Dead staining, and with the following antibodies: APC-anti-CD45 (clone A20, eBioscience); PE-Fluor 610-anti-F4/80 (clone BM8, eBioscience). Cells were processed by MACSQuantTM flow cytometer (Miltenyi Biotec); 40000 events were recorded, and compensation was performed using beads (BD Biosciences Pharmingen, San Diego, CA). Data were analysed by FlowJo Software (FlowJo, LLC).

**Immunoblot**

Standard protocol was used to perform Western blot analysis (Bio-Rad Laboratories). Briefly, Kup5 cells were isolated from culture plates by scraping and lysed in M-Per (Thermo Fisher Scientific, 78501) supplemented with protease and phosphatase inhibitors (Thermo Fisher Scientific, 78443). The Bradford assay (Bio-Rad Laboratories, 5000006) was used for protein quantification; following denaturation of same amount of protein was performed at 95°C in Laemmli buffer. Resolution was run on SDS-PAGE followed by transfer to a polyvinylidene fluoride membrane (Sigma, GE10600023). Membrane was blocked in 5% skim milk and incubated overnight at 4°C with primary antibody. After 1 hour incubation with HRP-conjugated secondary antibody (Cell Signalling Technology), signal was developed by ECL Select Western Blotting Detection Reagent (Amersham, RPN2235). For nuclear protein extraction was used the standard *subcellular fractionation protocol* (abcam). Briefly, Kup5 cells were scraped in a fractionation buffer (HEPES 20mM, KCl 10 mM, MgCl2 2 mM, EDTA 1mM, EGTA 1mM) supplemented with 1mM DTT and phosphatase inhibitor cocktail. Cell suspension was passed 10 times through a 27 Gauge needle by using a 1 ml syringe and centrifugated at 720g for 5 minutes. The pellet, containing nuclei was resuspended in fractionation buffer and passed ten time through 25 Gauge needle. Centrifugation at 720g was repeated for 10 minutes and pellet resuspended in TBS with 0.1% SDS and homogenised by sonication.

The following antibodies were used: anti-phospho-p38 MAPK (Cell Signalling Technology), anti-p38 MAPK (Cell Signalling Technology), anti-phospho-IKKα/β (Cell Signalling Technology), anti-IKKα (Cell Signalling Technology), anti-IKKβ (Cell Signalling Technology), anti-phospho-IkBα (Cell Signalling Technology), anti-IkBα (Cell Signalling Technology), anti-NFkB (p65) (Cell Signalling Technology) with anti-ß-actin (Sigma) as loading control.


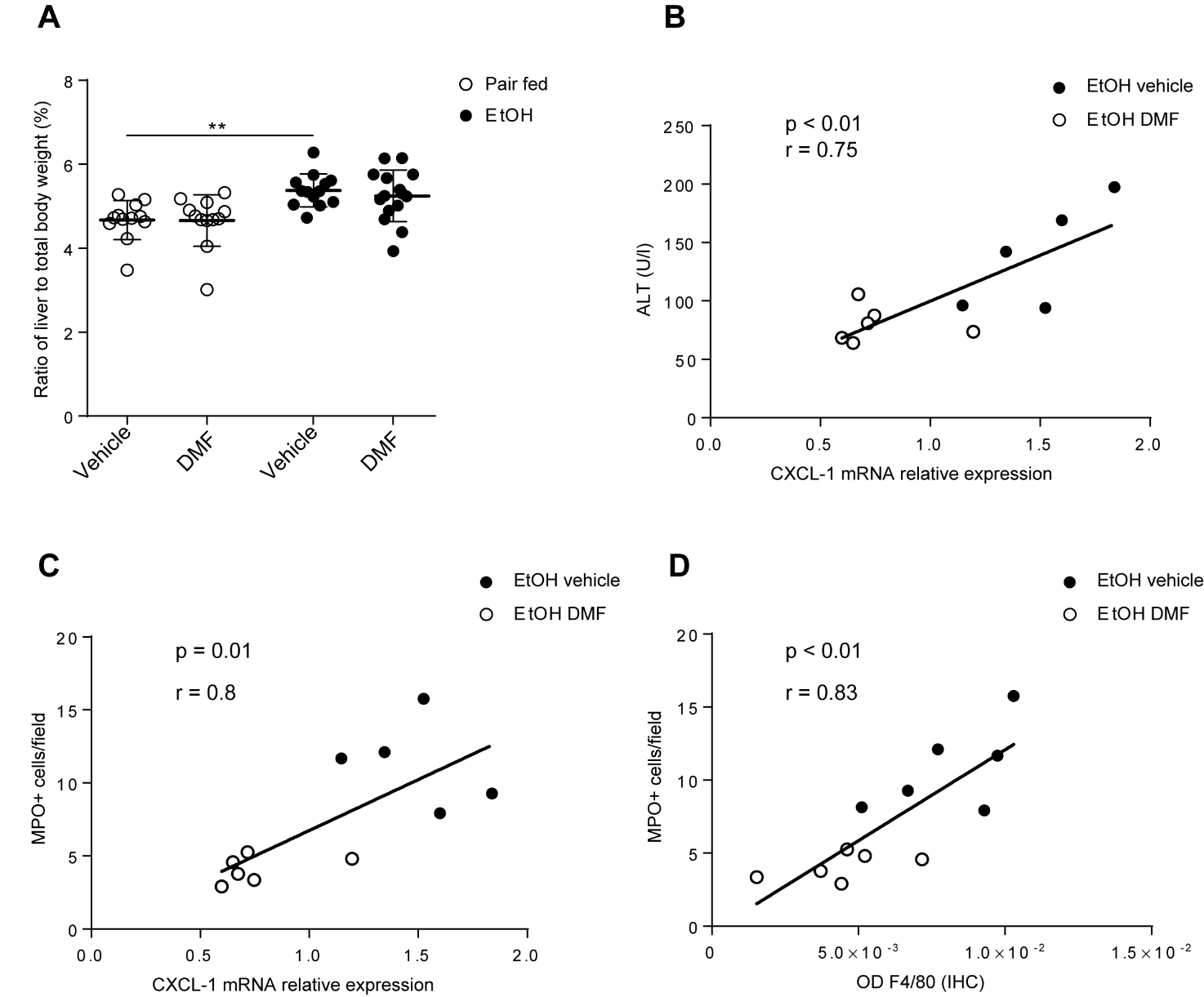


**Supplementary Fig. 1 Hepatic inflammation and liver injury. (A)** Ratio of Liver weight to total body weight (n=12-15 per group). **(B)** Correlation between CXCL-1 mRNA expression and serum ALT in overall ethanol fed mice (n=11). **(C)**  Correlation between CXCL-1 mRNA expression and MPO+cells/field at immunostaining in overall ethanol fed mice (n=11). **(D)** Correlation between amount of neutrophils and KCs determined by immunoreactivity at MPO and F4/80 respectively in overall ethanol fed mice (n=12). For (A) data are expressed in mean ± SEM; *p<0.05; **p<0.01; ***p<0.001 according to one-Way ANOVA followed by post hoc analysis (Bonferroni test). For (B-D) Spearman’s correlation test is used.


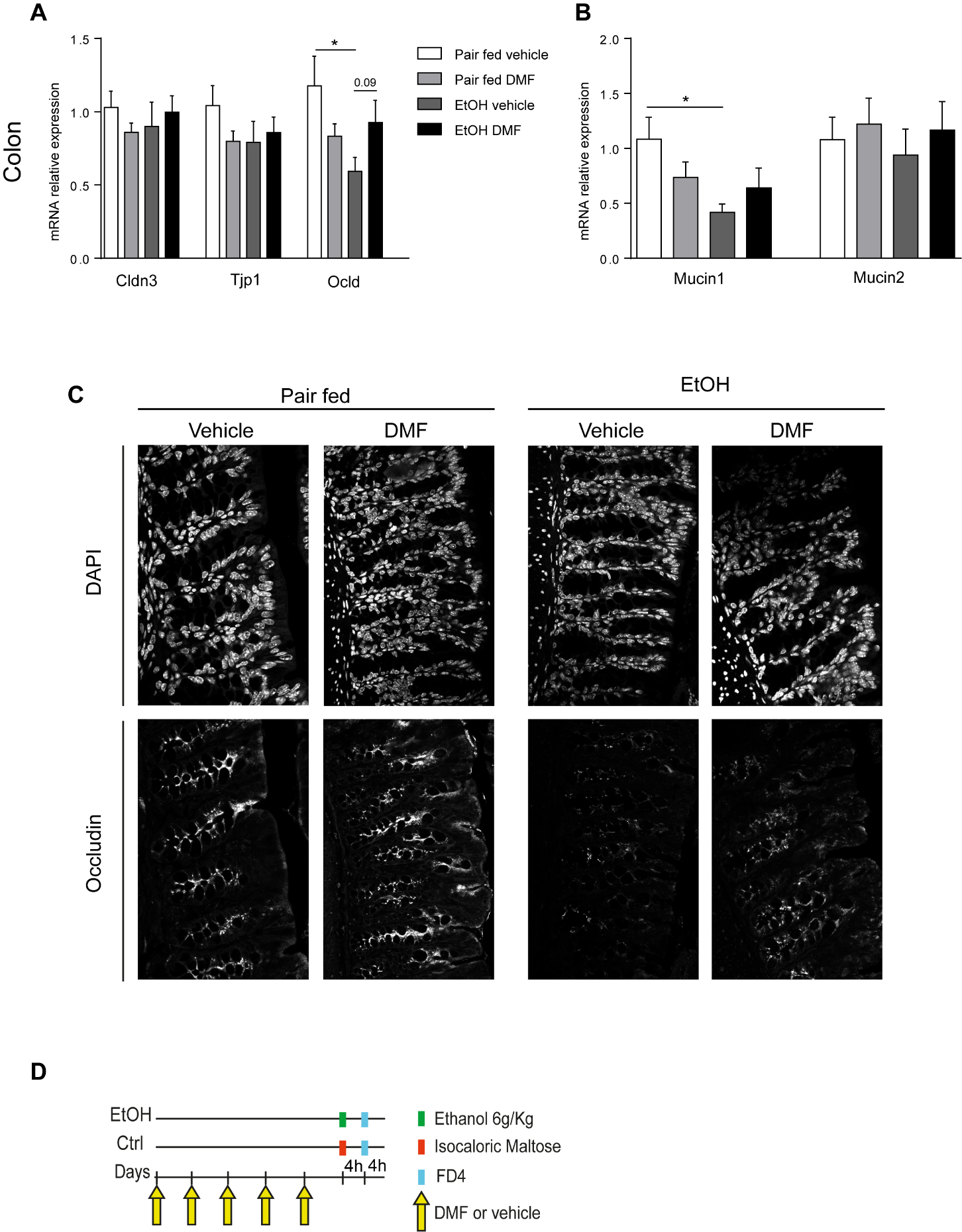


**Supplementary Fig. 2 Ethanol disrupts intestinal epithelial barrier. (A)** Colon genetic expression of tight junction proteins (Cldn3; Tjp1; Ocld) fold over *Pair fed vehicle*, determined by qPCR (n=5-6 per group). **(B)** Colon genetic expression of Mucin 1 and Mucin2 fold over *Pair fed vehicle*, determined by qPCR (n=5-6 per group). **(C)** Representative pictures of occludin immunoreactivity captured with confocal microscope, shown in grey scale (see Fig. 3 for merged picture). **(D)** Schematic representation of intestinal permeability assay. Data are expressed in mean ± SEM; *p<0.05; **p<0.01; ***p<0.001 according to one-Way ANOVA followed by post hoc analysis (Bonferroni test).


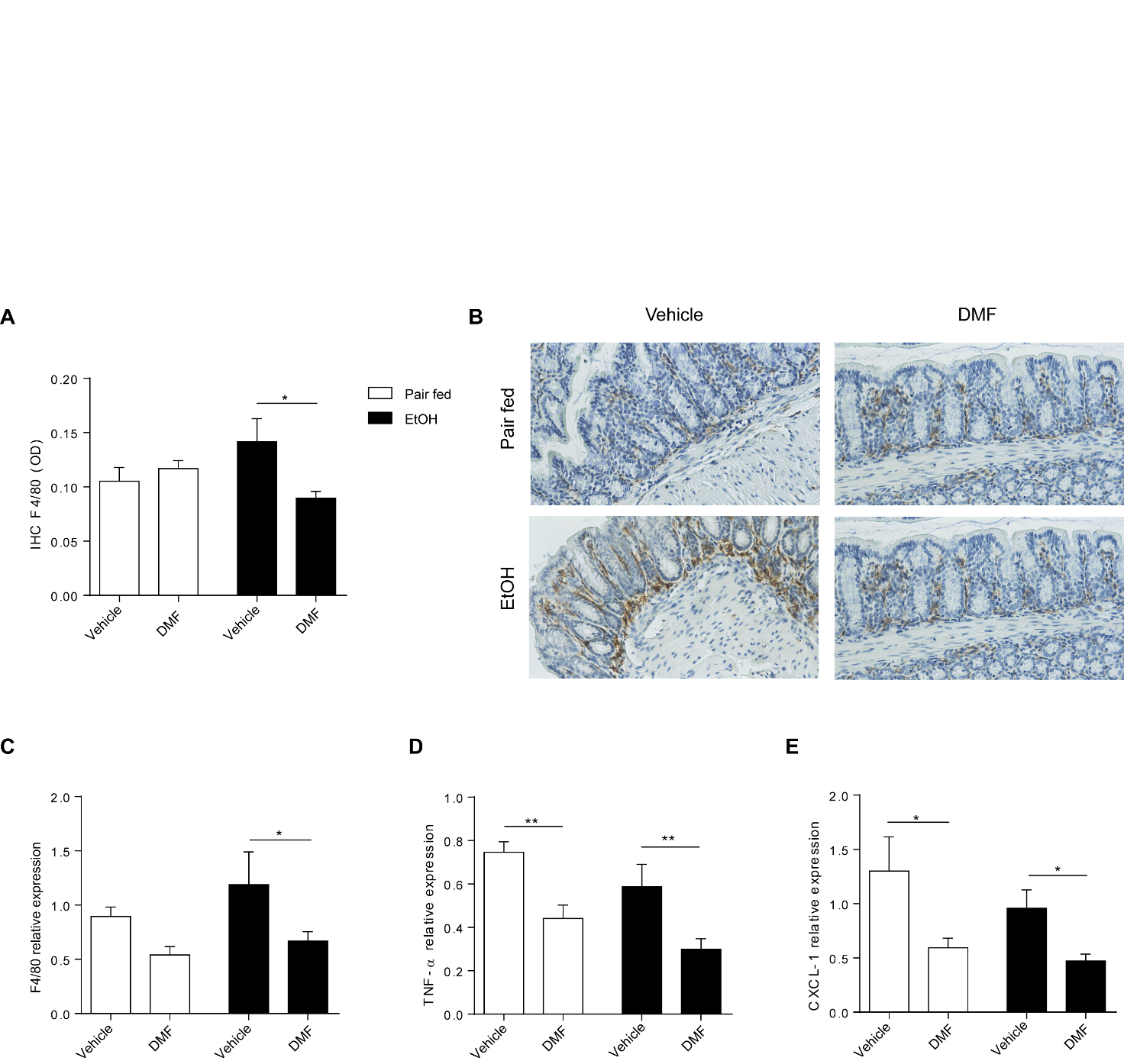


**Supplementary Fig. 3 DMF impacts on gut immune system. (A and B)** Representative pictures and quantification of macrophages in lamina propria determined by immunoreactivity to F4/80 (brown; Pair fed groups=n4; EtOH groups=n5). **(C – E)** Colon genetic expression of F4/80, TNF-α and CXCL-1 fold over *Pair fed vehicle*, determined by qPCR (n=5-6 per group). Data are expressed in mean ± SEM; *p<0.05; **p<0.01; ***p<0.001 according to one-Way ANOVA followed by post hoc analysis (Bonferroni test).


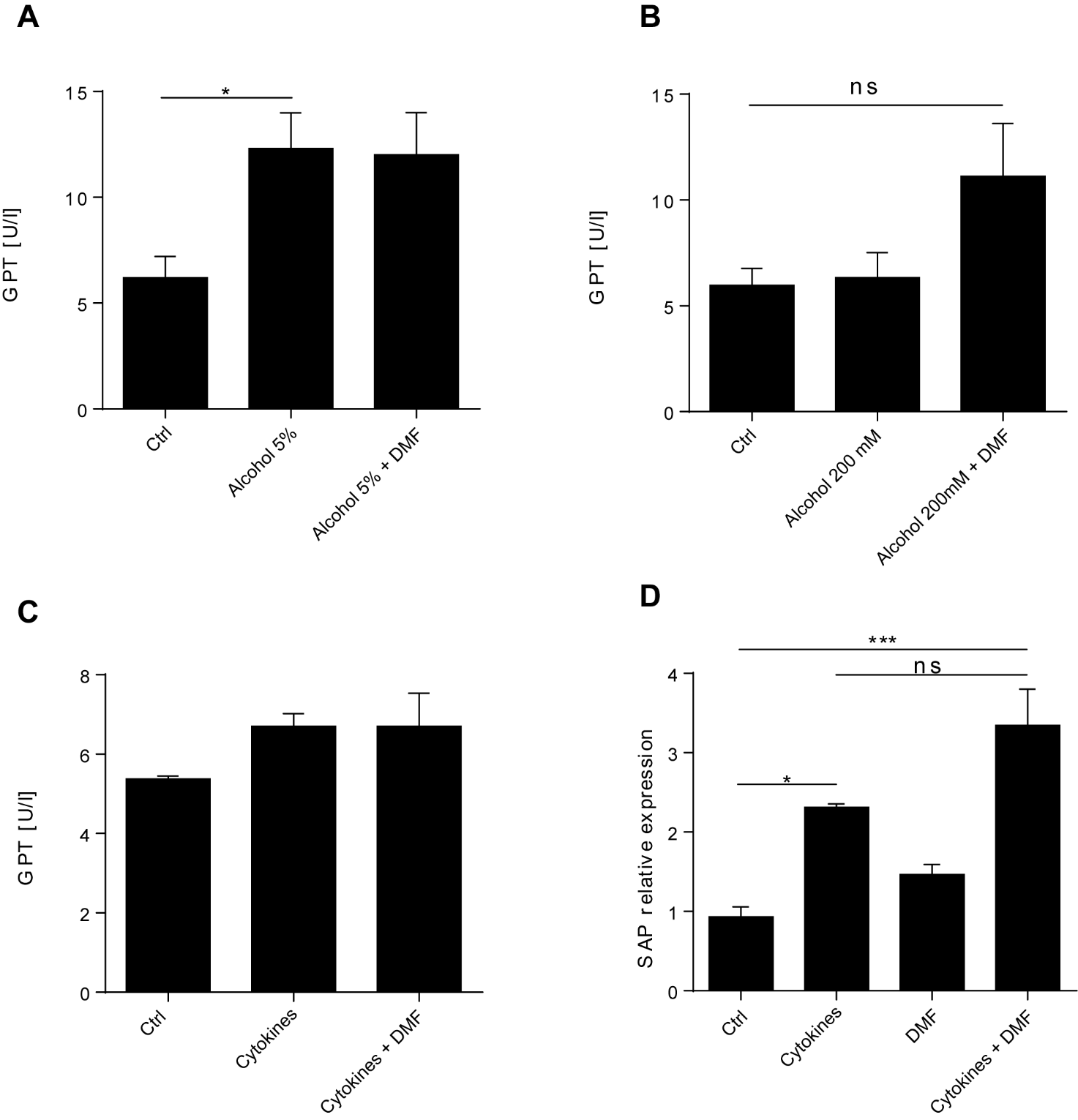


**Supplementary Fig.4 DMF does not protect hepatocytes (FL83B). (A and B)** GPT levels in supernatant of murine hepatocytes (FL83B) challenged with ethanol (high concentration: 5%; low concentration: 200 mM) and treated with DMF (50 µM) for 24 hours. **(C)** GPT levels in supernatant of murine hepatocytes (FL83B) challenged with a cytokine cocktail (TNF-α at 20 ng/ml and IL-1β at 10 ng/ml) and treated with DMF (50 µM) for 24 hours. **(D)** expression of *serum amyloid P* (SAP) determined by qPCR in murine hepatocytes (FL83B) challenged with a cytokine cocktail (TNF-α at 20 ng/ml and IL-1β at 10 ng/ml) and treated with DMF (50 µM) for 4 hours. Data are expressed in mean ± SEM; *p<0.05; **p<0.01; ***p<0.001 according to one-Way ANOVA followed by post hoc analysis (Bonferroni test).

Reference List

[1] Grander C, Adolph TE, Wieser V, Lowe P, Wrzosek L, Gyongyosi B et al: Recovery of ethanol-induced Akkermansia muciniphila depletion ameliorates alcoholic liver disease. *Gut* 2018, 67(5):891-901.
